# Histogram Analysis of Apparent Diffusion Coefficient in Differentiating Pancreatic Adenocarcinoma and Neuroendocrine Tumor

**DOI:** 10.1097/MD.0000000000002574

**Published:** 2016-01-29

**Authors:** Toshikazu Shindo, Yoshihiko Fukukura, Tomokazu Umanodan, Koji Takumi, Hiroto Hakamada, Masanori Nakajo, Aya Umanodan, Junichi Ideue, Kiyohisa Kamimura, Takashi Yoshiura

**Affiliations:** From the Department of Radiology, Kagoshima University Graduate School of Medical and Dental Sciences, Sakuragaoka, Kagoshima City, Japan.

## Abstract

The aim of this study was to investigate whether histogram analysis in diffusion-weighted (DW) magnetic resonance imaging (MRI) can help differentiate pancreatic adenocarcinomas from neuroendocrine tumors.

Sixty-four patients with histologically confirmed 53 pancreatic adenocarcinomas or 19 neuroendocrine tumors underwent DW MRI. We evaluated the pixel distribution histogram parameters (mean, skewness, kurtosis, and entropy) of the apparent diffusion coefficient (ADC) values derived from *b*-values of 0 and 200 (ADC_200_), 0 and 400 (ADC_400_), or 0 and 800 (ADC_800_) s/mm^2^. Histogram parameters were compared between pancreatic adenocarcinomas and neuroendocrine tumors, and the diagnostic performance was evaluated by using receiver operating characteristic (ROC) analysis.

The mean ADC_200_ and ADC_400_ were significantly higher in neuroendocrine tumors than in pancreatic adenocarcinomas (*P* = 0.001 and *P* = 0.019, respectively). Pancreatic adenocarcinomas showed significantly higher skewness and kurtosis on ADC_400_ (*P* = 0.007 and *P* = 0.001, respectively) and ADC_800_ (*P* = 0.001 and *P* = 0.001, respectively). With all *b*-value combinations, the entropy of ADC values was significantly higher in pancreatic adenocarcinomas (*P* < 0.001 for ADC_200_; *P* = 0.001 for ADC_400_; *P* < 0.001 for ADC_800_), and showed the highest area under the ROC curve for diagnosing adenocarcinomas (0.77 for ADC_200_, 0.76 for ADC_400_, and 0.78 for ADC_800_).

ADC histogram analysis of DW MRI can help differentiate pancreatic adenocarcinomas from neuroendocrine tumors.

## INTRODUCTION

Pancreas cancer is the seventh leading cause of cancer deaths across the world.^1,2^ Pancreatic adenocarcinomas account for 90% of cancers of the pancreas.^2^ Neuroendocrine tumors are the second most common malignant neoplasm of the pancreas, with an annual incidence of 2.2 per 1,000,000 individuals.^3^ Accurate differentiation between pancreatic adenocarcinomas and neuroendocrine tumors is of critical importance, because pancreatic adenocarcinomas remain a highly lethal disease with a 5-year survival rate of less than 10%,^1,2,4^ whereas neuroendocrine tumors generally have a slow-growing malignant profile and a better prognosis.^5^ However, despite great technical advances in imaging, such as the development of multidetector computed tomography (CT) and magnetic resonance imaging (MRI), assigning the correct diagnosis of pancreatic adenocarcinomas and neuroendocrine tumors still remains challenging owing to the overlapping imaging features of these tumors.^6–9^

Diffusion-weighted (DW) MRI has been used for various aspects of the evaluation of pancreas lesions, including detection, diagnosis, and prediction of patient prognosis, as it can be performed relatively quickly and has excellent contrast resolution without the administration of contrast agents.^10–14^ DW imaging reflects changes in water mobility caused by interactions with cell membranes, macromolecules, and alterations to the tissue environment, thus providing qualitative and quantitative information pertinent to tissue structure; it does so based on measurement of the thermally induced diffusivity of water molecules shown by the apparent diffusion coefficient (ADC), which can be displayed as an ADC map over the area of data acquisition.

Although most studies of cancer have been conducted with mean ADC values from tumor regions of interest (ROIs), it is being increasingly recognized that the heterogeneity of diffusion in the tumor region can be examined using ADC histogram analysis based on pixel distribution. ADC histogram analysis is a reproducible technique,^15^ and has been reported to be particularly effective in reflecting microstructure of tumors of the head,^16–18^ neck,^19,20^ uterus,^21,22^ bladder,^23^ and ovary.^24^ However, the ability of DW imaging to discriminate between pancreatic adenocarcinoma and neuroendocrine tumor is less established.^12,25,26^ Moreover, there have been no reports regarding the utility of ADC histogram analysis in differentiating pancreatic adenocarcinomas from neuroendocrine tumors.

Therefore, the purpose of this study was to investigate whether histogram analysis in DW MRI can help differentiate pancreatic adenocarcinomas from neuroendocrine tumors.

## METHODS

### Study Population

Institutional ethics review board approval was obtained and informed consent was waived for this retrospective study. Between April 2012 and October 2013, a total of 81 patients who were diagnosed with pancreatic adenocarcinomas or neuroendocrine tumors were retrospectively identified through a review of clinical charts and records in the Department of Human Pathology. Within this group, 71 patients with 60 pancreatic adenocarcinomas or 24 neuroendocrine tumors were considered for inclusion in this retrospective study; all of these patients had undergone DW MRI, which has been systematically used as part of our MRI protocol for pancreatobiliary diseases since April 2012. We excluded 5 patients with pancreatic adenocarcinomas who had received radiotherapy and/or chemotherapy before MRI, because these conditions might alter the water mobility of the measurement. Two pancreatic adenocarcinomas and 5 neuroendocrine tumors also were excluded due to severe image degradation resulting from a susceptibility artefact and a too-small tumor size (<10 mm) to evaluate on DW imaging, respectively, because this might have affected the evaluation of ADC values. The final study population of pancreatic adenocarcinomas contained 53 patients (31 men, 22 women; mean age, 68.6 years; range, 42–83 years). Lesion sizes ranged from 11.7 to 73.0 mm (mean, 31.0 mm) (Table 1). Nineteen neuroendocrine tumors (mean size, 25.7 mm; range, 10.0–83.5 mm) in 11 patients (3 men, 8 women; mean age, 52.8 years; range, 26–80 years) were finally included in this study. No statistically significant difference in the tumor size (*P* = 0.20, 2-tailed Student *t* test) or location (*P* = 0.60, Fisher exact test) was observed between pancreatic adenocarcinomas and neuroendocrine tumors. The final diagnoses of pancreatic adenocarcinoma and neuroendocrine tumor were confirmed by a pathologist with 20 years of experience in pathological evaluation following surgical resection (n = 16) or percutaneous ultrasonography-guided needle biopsy (n = 48).

**TABLE 1:**
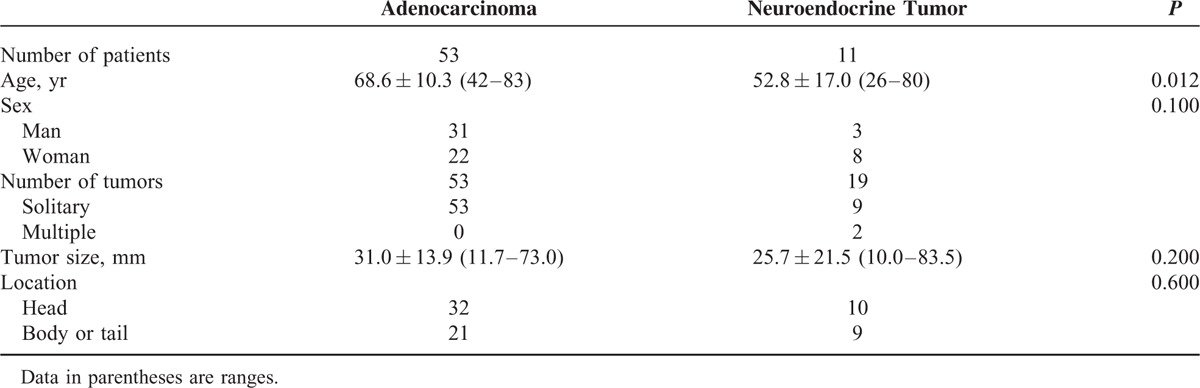
Characteristics of Patients With Pancreatic Adenocarcinomas or Neuroendocrine Tumors of the Pancreas

### MR Imaging Protocols

The MRI examination was performed using a 3.0–T system (Ingenia 3.0 T; Philips Healthcare, Best, The Netherlands) equipped with a dStream Torso coil.

The DW images with 4 *b*-values (0, 200, 400, and 800 s/mm^2^) were acquired in the transverse plane by respiratory-triggered single-shot echo-planar imaging using the navigator-echo technique. A section thickness of 5 mm was applied, and the intersection gap was 0.5 mm to cover the whole liver and right kidney. Pulse sequence parameters used were as follows: repetition time, depending on respiratory intervals; echo time, 60 ms; flip angle, 90^o^; field of view, 350 mm; matrix, 60 × 112; number of excitations, 2 for *b*-values of 0, 200, and 400 s/mm^2^ or 4 for *b*-value of 800 s/mm^2^; sensitivity encoding acceleration factor, 4; motion-probing gradient pulse, 3 orthogonal diffusion directions; and acquisition time, approximately 3 to 4 min. Frequency-selective fat saturation was used to reduce chemical shift artefacts.

The clinical MRI study also included T1-weighted dual gradient-echo and respiratory-triggered T2-weighted turbo spin echo with fat-suppression imaging (n = 64). Gadoxetic acid-enhanced fat-suppressed T1-weighted images were also obtained to evaluate liver metastases (n = 59).

### Image Analysis

**FIGURE 1:**
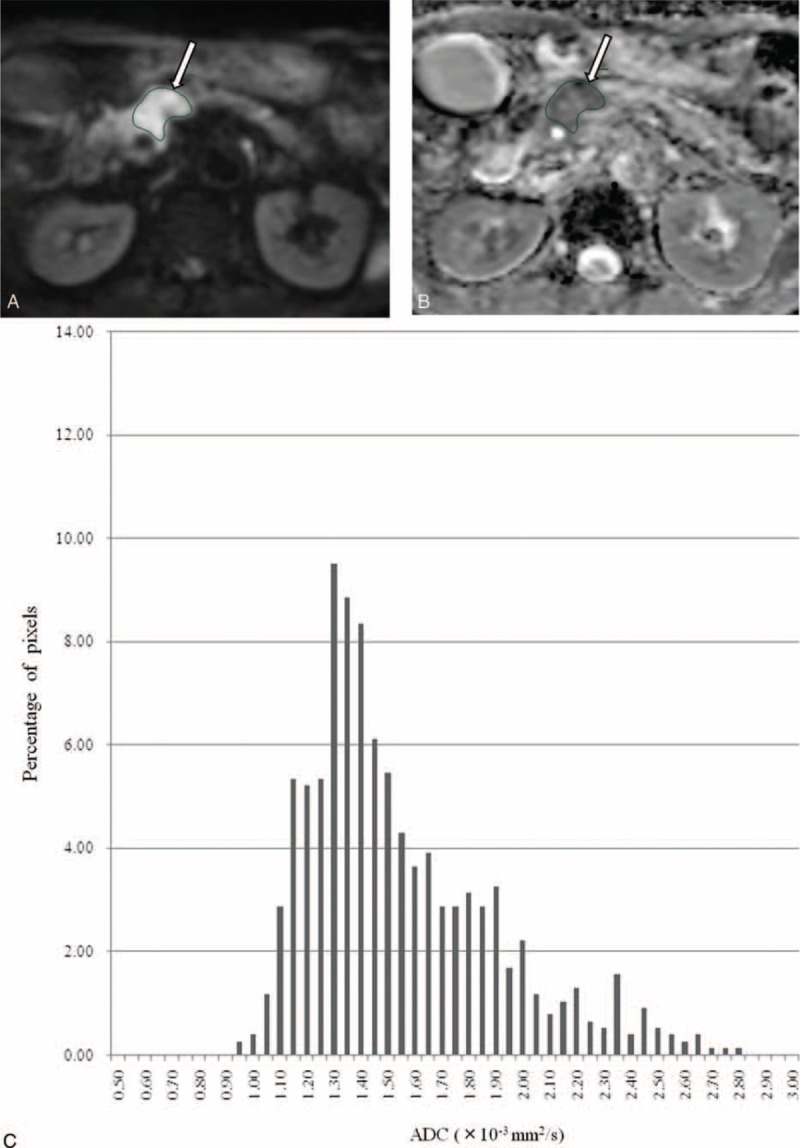
A 59-year-old man with pancreatic adenocarcinoma in the pancreas head. Tumor region of interest (ROI) was localized on (A) diffusion-weighted image and (B) apparent diffusion coefficient map derived from *b*-values of 0 and 800 s/mm^2^ (*arrow*). (C) Corresponding ADC histogram of the tumor within the ROI demonstrates wide range (mean, 1.52; kurtosis, 0.70; skewness, 1.06; entropy, 2.70), indicating the heterogeneous tissue components within pancreatic adenocarcinoma.

**FIGURE 2:**
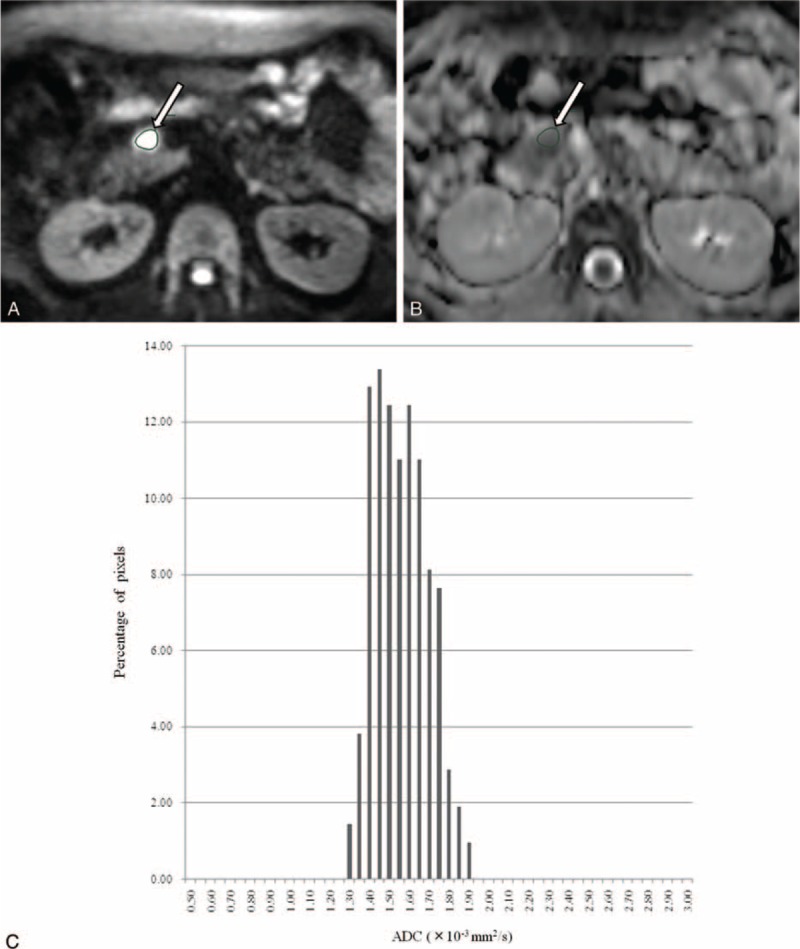
A 49-year-old woman with neuroendocrine tumor in the pancreas head. Tumor region of interest (ROI) was localized on (A) diffusion-weighted image and (B) apparent diffusion coefficient map derived from *b*-values of 0 and 800 s/mm^2^ (*arrow*). (C) Corresponding ADC histogram of the tumor within the ROI demonstrates narrow range (mean, 1.54; kurtosis, −0.71; skewness, 0.27; entropy, 2.21), indicating the homogeneous tissue components within neuroendocrine tumor.

DW images were digitally transferred to SYNAPSE VINCENT software (FUJIFILM Medical, Tokyo, Japan). ADC maps at either of a pair of *b*-values—0 and 200 (ADC_200_), 0 and 400 (ADC_400_), and 0 and 800 s/mm^2^ (ADC_800_)—for histogram analysis were calculated on a voxel-by-voxel basis. The following parameters were derived from the ADC histograms: mean; skewness, which is a measure of the degree of asymmetry of a distribution; kurtosis, which is the degree of peakedness of a distribution; and entropy, which is a textural-based measure of the variation and predictability of individual values in the overall histogram distribution of values across the lesion. In this study, skewness, kurtosis, and entropy are defined as *E*[(*x* − *μ*)]^3^/*σ*^3^, *E*[(*x* − *μ*)]^4^/*σ*^4^, and Σ(−*p*_*i*_)log(*p*_*i*_), respectively, where *E* is the expected value, *μ* is the mean of *x*, *σ* is the standard deviation of *x,* and *p*_*i*_ represents the probability of ADC value in the image and is calculated by dividing the number of voxels in each ADC value by the number of voxels in all ADC values. To obtain histograms of ADCs, 2 independent radiologists (TS and YF, with 15 and 22 years of experience, respectively), who had no knowledge of the final pathological results, manually drew around the pancreatic tumor including areas of necrosis on each map with the maximum diameter of tumor (mean size, 487 mm^2^; range, 24–1699 mm^2^) (Figures 1 and 2). The ROI was first localized on DW images with reference to T1-weighted, T2-weighted, gadoxetic acid-enhanced T1-weighted images, and/or 3-phase enhanced CT; and the ROI was mirrored to each ADC map (ADC_200_, ADC_400_, and ADC_800_).

### Statistical Analysis

Statistical analyses were performed using SPSS for Windows version 22.0 software (SPSS Inc, Chicago, IL). Intraclass correlation coefficient (ICC) was calculated to evaluate interobserver agreement of all histogram parameters. Values of *P* < 0.05 were considered to indicate a significant difference.

Histogram parameters (mean, skewness, kurtosis, and entropy) of ADCs (ADC_200_, ADC_400_, and ADC_800_) were compared between pancreatic adenocarcinomas and neuroendocrine tumors by using the Mann–Whitney *U* test. Receiver operating characteristic (ROC) curve analyses for histogram parameters of ADC_200_, ADC_400_, and ADC_800_ were performed to evaluate diagnosing performance to differentiate pancreatic adenocarcinomas from neuroendocrine tumors. Diagnostic performance was determined by calculating the area under the ROC curve (AUC). Sensitivity and specificity were calculated by using a threshold criterion, which was determined by the largest Yoden index (the sum of sensitivity and specificity).^27^

## RESULTS

ADC histogram results are summarized in Table 2. Interobserver agreements were good to excellent with ICCs of 0.71 to 0.99. The mean ADC_200_ (*P* = 0.001) and ADC_400_ (*P* = 0.019) were significantly higher in neuroendocrine tumors than in pancreatic adenocarcinomas, although no significant difference was obtained with ADC_800_ (*P* = 0.59). Histograms of ADC_400_ and ADC_800_ demonstrated wider range in pancreatic adenocarcinomas than in neuroendocrine tumors (Figures 1 and 2). Skewness and kurtosis of ADC_400_ (*P* = 0.007 and *P* = 0.001, respectively) and ADC_800_ (*P* = 0.001 and *P* = 0.001, respectively) were significantly higher in pancreatic adenocarcinomas than in neuroendocrine tumors, although no significant difference was obtained with ADC_200_ (*P* = 0.10 and *P* = 0.07, respectively). With all *b*-value combinations (*P* < 0.001 for ADC_200_; *P* = 0.001 for ADC_400_; *P* < 0.001 for ADC_800_), the entropy of ADC values was significantly higher in pancreatic adenocarcinomas than in neuroendocrine tumors.

**TABLE 2:**
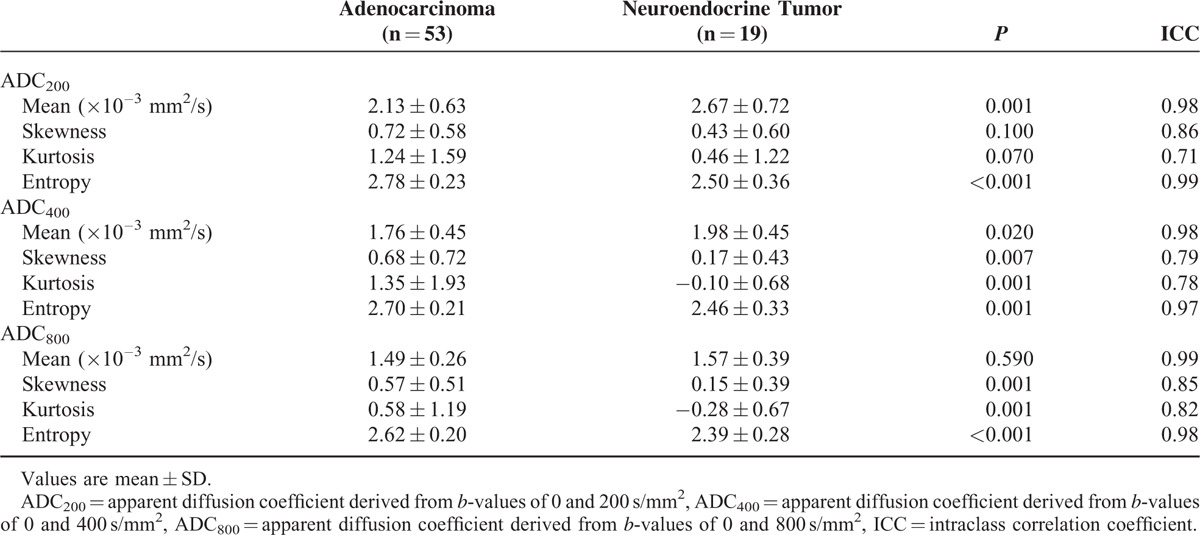
Histogram Analysis of Apparent Diffusion Coefficient Values Derived From *b*-Values of 0 and 200, 0 and 400, and 0 and 800 s/mm^2^

ROC analyses and diagnostic performances for differentiating pancreatic adenocarcinomas from neuroendocrine tumors are shown in Table 3. The entropy showed the highest AUC among ADC histogram parameters, with a sensitivity of 73.6% and specificity of 79.0% with ADC_200_, a sensitivity of 71.7% and specificity of 79.0% with ADC_400_, and a sensitivity of 54.7% and specificity of 89.5% with ADC_800_.

**TABLE 3:**
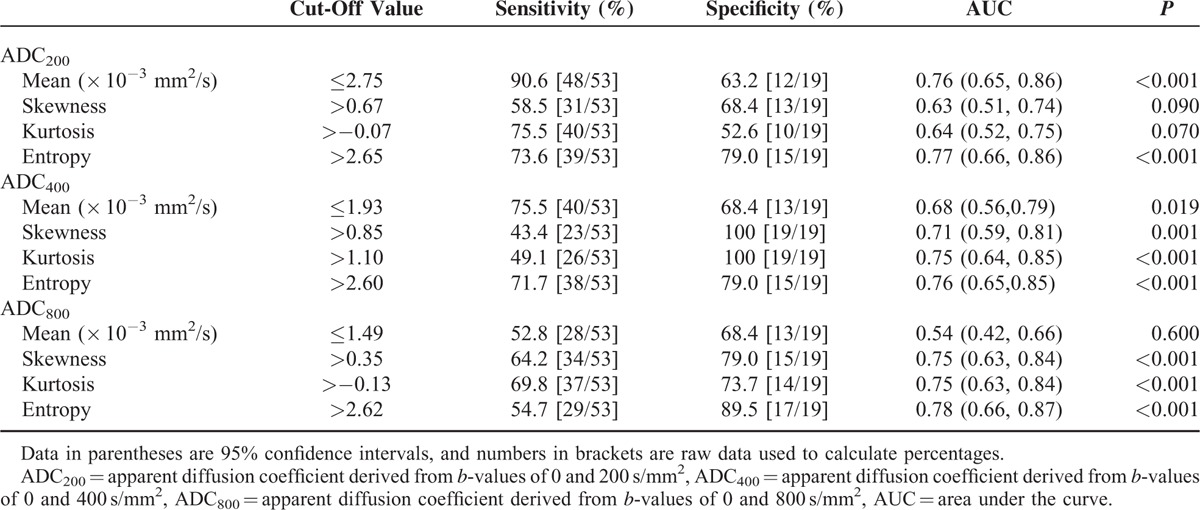
Receiver Operating Characteristic Analysis of Histogram Parameters in Differentiating Pancreatic Adenocarcinomas From Neuroendocrine Tumors

## DISCUSSION

Among the ADC histogram parameters, in our study, the entropy of ADC values was most useful for distinguishing pancreatic adenocarcinomas from neuroendocrine tumors. In clinical practice, dynamic CT or MRI is frequently used to demonstrate this difference in contrast enhancement and vascular encasement between the 2 diseases. However, these findings sometimes overlap, thereby making differential diagnosis difficult.^6–9^ Therefore, we believe that the entropy of ADC values might add helpful information in differentiating pancreatic adenocarcinomas from neuroendocrine tumors, especially in patients with contraindication to contrast agents (ie, contrast agent allergy, severe renal failure) or with focal pancreatic lesions showing atypical findings at dynamic CT or MRI.

Previously, researchers have explored the value of DW imaging in the characterization of several pancreatic lesions, such as pancreatic adenocarcinoma,^11,12,25,26,28^ autoimmune or chronic pancreatitis,^12,25,26,28^ neuroendocrine tumor,^12,25,26,29^ and cystic lesions.^30,31^ However, the ability of DW imaging to discriminate between pancreatic adenocarcinoma and neuroendocrine tumor has been limited.^12,25,26,32^ Lee et al^12^ reported that there was no significant difference in mean ADC values obtained by using *b*-values of 0 and 500 or 0 and 1000 s/mm^2^ between pancreatic adenocarcinomas and neuroendocrine tumors. Yao et al^32^ reported no significant difference in mean ADC by using values of 0 and 600 s/mm^2^ between pancreatic adenocarcinomas and neuroendocrine tumors. Concia et al^26^ reported that mean ADC values obtained by using of 0 and 50 s/mm^2^ of neuroendocrine tumors were significantly higher than those of pancreatic adenocarcinomas, whereas there was no significant difference in mean ADC values using *b*-values of 0 and 800 s/mm^2^. The choice of *b*-value plays important role for ADC value, because ADC represents perfusion as well as molecular diffusion. In principle, applying lower *b*-values increases perfusion and decreases diffusion effects.^33^ In our study, mean ADC values using *b*-values of 0 and 200 (ADC_200_), and 0 and 400 s/mm^2^ (ADC_400_) were higher in neuroendocrine tumors than in pancreatic adenocarcinomas, but no significant difference was obtained using *b*-values of 0 and 800 s/mm^2^ (ADC_800_). This result is supported by a previous study, which showed that the perfusion-related parameters of intravoxel incoherent motion DW imaging might be a more important factor than true molecular diffusion in differentiating pancreatic adenocarcinoma and neuroendocrine tumor.^25^

We used histogram analysis to evaluate not only mean ADCs, but also skewness, kurtosis, and entropy, which reflect the distribution of ADC values. There have been no reports assessing the usefulness of ADC histogram analysis for differentiation between pancreatic adenocarcinoma and neuroendocrine tumor. In our study, skewness of ADC_400_ and ADC_800_ was significantly higher in pancreatic adenocarcinomas than in neuroendocrine tumors. Skewness reflects the asymmetry of the ADC distribution. A skewness of zero indicates that ADC values within the lesion are evenly distributed above and below the mean ADC value. A positive skewness indicates that most of the voxels contain an ADC value less than the mean and long tail of the curve is more toward the right side. A negative skewness indicates the reverse of a positive skewness. Therefore, pancreatic adenocarcinomas are considered to contain more voxels with ADC values less than the mean, compared with neuroendocrine tumors.

Pancreatic adenocarcinoma had a higher kurtosis than neuroendocrine tumor with ADC_400_ and ADC_800_. Kurtosis reflects the peakedness and heaviness of the tails of the distribution of ADC values. A histogram with normal distribution has a kurtosis of zero; a positive kurtosis indicates a relatively sharper peak and wider tails; and a negative kurtosis indicates a relatively rounded peak and shorter tails. Therefore, a higher kurtosis of ADC in pancreatic adenocarcinoma could be expected as it is more heterogeneous.

Entropy describes the variation in ADC histogram. In our study, significantly higher entropy of ADC with every *b*-value combination was found in pancreatic adenocarcinoma, which reflects higher heterogeneity of pancreatic adenocarcinoma compared with neuroendocrine tumor. The entropy of ADC with every *b*-value combination showed the highest AUC with 0.77 for ADC_200_, 0.76 for ADC_400_, and 0.78 for ADC_800_. Thus, the entropy of ADC may be more helpful than the mean ADC and other histogram parameters for differentiating pancreatic adenocarcinomas from neuroendocrine tumors.

Fibrosis and mucin within tumors are common histopathological features of pancreatic adenocarcinoma. It has been reported that the voxel with low ADC value is well correlated with highly cellular components within the tumor, whereas the higher frequency of voxels with high ADC values reflects mucin or necrotic components.^18,21,34^ The loose fibrosis within pancreatic adenocarcinoma may account for the freedom of diffusion of water molecules and high ADC values, while dense fibrosis may restrict water diffusion, resulting in lower ADC values.^13^ Therefore, the ADC histogram parameters can reflect pathologic features of pancreatic adenocarcinoma and neuroendocrine tumor. A higher skewness, kurtosis, and entropy in adenocarcinomas might be attributed to the heterogeneity due to the abundant fibrosis or mucin within the tumors. For neuroendocrine tumors in which tumor cell density is more homogenous, the distribution of ADC values was closer to uniform across the range.

Some limitations in our study must be considered. First, it was a retrospective study with a small population size, and the number of patients for each tumor was unbalanced. Second, it was difficult to evaluate the correlation of ADC histogram analysis with pathologic features such as cellular component, mucin, fibrosis, and necrosis, because the number of patients with surgical resection (10 pancreatic adenocarcinomas and 6 neuroendocrine tumors) was limited. Further studies regarding correlation between the ADC histogram parameters and histopathological features are warranted. Third, our histogram analysis was based on the largest representative single cross-sectional area of tumor, thus excluding potential information from the rest of the volume. Previous researchers used the whole volume on all slices encompassing the entire tumor for histogram analyses, a methodology that might be more objective and better reflect the heterogeneity of the lesion.^18,19,21,34^ However, the entire tumor histogram analysis is cumbersome and may not be practical in daily clinical practice. Moreover, our main concerns were that including the several first and last slices would carry disadvantageous influences of partial volume effect, because tumors were relatively small, although small tumors (<10 mm) were excluded in our study. Skewness and kurtosis of ADC with *b*-values of 0 and 400 and 0 and 800 s/mm^2^, and entropy of ADC values with every *b*-value combination were significantly higher in pancreatic adenocarcinomas than in neuroendocrine tumors. Interobserver agreements were 0.71 to 0.99. Therefore, we believe that our ADC histogram analysis based on ROI measurement was a reproducible method and has the potential to differentiate pancreatic adenocarcinomas from neuroendocrine tumors. However, further investigation should be necessary to clarify whether the use of the largest representative single cross-sectional area of tumor is objective and can reflect the heterogeneity of the lesion in comparison with that of the whole tumor volume.

In conclusion, this study was focused on the potential of ADC histogram analysis in DW imaging to differentiate pancreatic adenocarcinoma from neuroendocrine tumor. Skewness and kurtosis of ADC with *b*-values of 0 and 400 and 0 and 800 s/mm^2^, and entropy of ADC values with every *b*-value combination were significantly higher in pancreatic adenocarcinomas than in neuroendocrine tumors. The entropy of ADC values showed the highest AUC for diagnosing pancreatic adenocarcinoma among the ADC histogram parameters. Histogram analysis of ADC maps might be helpful in differentiating pancreatic adenocarcinomas from neuroendocrine tumors.
